# Event-Related Potentials Elicited by Pre-Attentive Emotional Changes in Temporal Context

**DOI:** 10.1371/journal.pone.0063703

**Published:** 2013-05-09

**Authors:** Tomomi Fujimura, Kazuo Okanoya

**Affiliations:** 1 Okanoya Emotional Information Project, Exploratory Research for Advanced Technology (ERATO), Japan Science and Technology Agency, Wako, Saitama, Japan; 2 Emotional Information Joint Research Laboratory, Brain Science Institute, RIKEN, Wako, Saitama, Japan; 3 Department of Life Sciences, Graduate School of Arts and Sciences, The University of Tokyo, Meguro, Tokyo, Japan; University of Rome, Italy

## Abstract

The ability to detect emotional change in the environment is essential for adaptive behavior. The current study investigated whether event-related potentials (ERPs) can reflect emotional change in a visual sequence. To assess pre-attentive processing, we examined visual mismatch negativity (vMMN): the negative potentials elicited by a deviant (infrequent) stimulus embedded in a sequence of standard (frequent) stimuli. Participants in two experiments pre-attentively viewed visual sequences of Japanese kanji with different emotional connotations while ERPs were recorded. The visual sequence in Experiment 1 consisted of neutral standards and two types of emotional deviants with a strong and weak intensity. Although the results indicated that strongly emotional deviants elicited more occipital negativity than neutral standards, it was unclear whether these negativities were derived from emotional deviation in the sequence or from the emotional significance of the deviants themselves. In Experiment 2, the two identical emotional deviants were presented against different emotional standards. One type of deviants was emotionally incongruent with the standard and the other type of deviants was emotionally congruent with the standard. The results indicated that occipital negativities elicited by deviants resulted from perceptual changes in a visual sequence at a latency of 100–200 ms and from emotional changes at latencies of 200–260 ms. Contrary to the results of the ERP experiment, reaction times to deviants showed no effect of emotional context; negative stimuli were consistently detected more rapidly than were positive stimuli. Taken together, the results suggest that brain signals can reflect emotional change in a temporal context.

## Introduction

Emotional events often modulate attention and perception, resulting in the facilitation of cognitive processing and behavioral responses. Surface electroencephalographic recordings have revealed that, compared to neutral stimuli, emotional stimuli modulate electrophysiological event-related potential (ERP) components [1]. For example, affectively arousing pictures were shown to enhance an early posterior negativity (EPN) at a 150–300 ms latency [2], [3] and a late positive potential (LPP) at a 350–400 ms latency measured over centro-parietal areas [3], [4], [5]. Recent studies have also shown that EPN and LPP are also evoked when viewing emotional words [6], [7], [8], [9]. These components reflect selective attention and the motivational relevance of emotional stimuli.

As mentioned above, a majority of the existing ERP studies on emotion have demonstrated neural activity in response to a single emotional event. However, emotional events are dynamic and continuously change according to real-life situations. For example, emotional events may occur without notice in a mundane life, and good fortune may come following a run of bad luck. It is therefore important to detect emotional change within a continuous stream of stimuli to address an incoming emotional event effectively. A previous study found that ERP amplitudes were modulated by emotional change in context [10]. Those results showed greater LPP amplitudes when a very positive word was embedded within very negative words rather than within mildly positive words. This suggests that the LPPs reflected not only the emotional content of the target stimulus but also the emotional contrast between the target stimulus and its context. This is the evidence for neural processing of emotional change in a temporal context. However, the study assessed ERPs during an evaluative categorization of the emotional words, with the LPP indicative of a higher-order cognitive process. Therefore, it remains unclear whether emotional change can be automatically processed in the brain.

To assess the neural processing of an emotional change without the higher-order cognitive process, it is useful to focus on the visual mismatch negativity (vMMN) that is observed in the occipital area within the 140–400 ms interval [11], [12]. MMN is the negative-going wave elicited when a deviant stimulus violates the regularity created by a sequence of standard stimuli [13], [14]. Although MMN was originally studied in the auditory modality, there is increasing evidence supporting the relevance of MMN to the visual domain [15], [16]. vMMN can be observed even when the participants are not using the vMMN-related stimulus stream for the overt task [17], [18]. Hence, vMMN is considered to reflect pre-attentive, automatic processing of changes in a sequence. Given that automatic detection of emotional signals in the environment is crucial for adaptive behavior, it is possible that vMMN is produced by emotional deviants in a sequence of stimuli.

Four recent studies have demonstrated that the vMMN reflects emotional changes in a sequence of facial stimuli [19], [20], [21], [22]. In two the studies, emotional faces were presented as deviant stimuli in a series of neutral faces [19], [20]. Those results indicated that the emotional deviant faces elicited greater occipital negativity in ERPs than neutral standard faces at latencies of 150–180 ms and 280–320 ms [19] and at latencies of 110–360 ms and 120–430 ms [20]. In the other two studies, emotional faces were infrequently presented among faces showing an opposite valence (i.e., happy faces among fearful faces). The ERPs for the emotional faces that were presented as deviants were compared to the ERPs for the identical faces that were presented as standards [21], [22]. These latter two studies found that the vMMN for emotional faces occurred within the 170–360 ms interval at the posterior site, suggesting that the sequential regularity in the repetition of emotional faces was interrupted by deviant faces displaying an opposite valence. Cumulatively, the evidence suggests that vMMN can reflect emotional changes, including those from neutral to emotional stimuli and from negative to positive stimuli and vice versa.

However, it is possible that the emergence of vMMN in response to emotional change is specific to face-processing. Recent studies have demonstrated that socially emotional stimuli (e.g., facial expressions) and biologically emotional stimuli (e.g., sexual images and mutilation) produce different somatic responses [23] and have different effects on neural activity and memory [24]. To confirm that vMMN can serve as a proximal index for identifying emotional changes, emotional stimuli other than facial expressions should be examined. For stimuli, then, the present study used Japanese kanji, which have different emotional connotations. A number of previous studies have also used Japanese kanji [25], [26], [27]. Unlike words, Japanese kanji can represent an emotion with only a single character. Furthermore, as Japanese kanji consist of simple line drawings with less complexity than pictures or scenes, processing kanji demands fewer cognitive resources. Taken together, Japanese kanji are well suited for investigating pre-attentive emotional processing.

Additionally, previous studies of vMMN in facial expressions have focused on changes within an emotional category (i.e., neutral vs. happiness/fear or happiness/fear vs. fear/happiness). Each emotional category can be characterized by a degree of intensity, which is one of the fundamental factors in describing emotional circumstances [28]. To further the understanding of vMMN for emotional changes, Experiment 1 of our study explored whether the amplitude of the negativity elicited by emotional changes was modulated by the magnitude of emotional intensity that characterized the deviants among neutral standards. A previous vMMN study which used color as the change modality reported that the vMMN amplitude increased with greater physical differences between the standards and deviants [29]. Therefore, if vMMN truly reflects emotional change in a sequence, we expect that strongly emotional deviants elicit more negativity than weakly emotional deviants against neutral standards.

To clarify ERPs for emotional change in a sequence, the present study investigated the relationship between vMMN and EPN. EPN elicited by affectively arousing stimuli in the 150–300 ms interval [2] is topographically and temporally similar to vMMN. Thus, it is possible that negative-going waves in response to emotional deviants reflect both the emotional significance of the deviants and the emotional contrast between the deviants and the standards (i.e., emotional change) in the sequence. To confirm that the observed negative shift was derived from emotional changes, a single character with an emotional connotation served as two types of deviants in Experiment 2. One was emotionally incongruent with the standard (i.e., positive deviants against negative standards) while the other was emotionally congruent with the standard (i.e., positive deviants against positive standards). If the negative shift for emotionally incongruent deviants was greater than that for emotionally congruent deviants, this component could be considered evoked by the emotional change in a sequence rather than by the emotional significance of the deviants.

In addition to investigating neural activity, the current study examined behavioral responses to pre-attentive changes in emotional stimuli within a sequence. We employed a paradigm identical to the one used in the ERP experiment. Previous behavioral studies have reported longer reaction times to negative stimuli than to neutral stimuli in circumstances where the emotional content of the stimuli is task-irrelevant [30], [31]. However, it remains unclear whether emotional change in a sequence modulates behavioral response. We hypothesized that if emotional changes modulate behavioral responses, then reaction times to emotionally incongruent deviants (i.e., a negative deviant among positive standards) should be faster than those to emotionally congruent deviants (i.e., a negative deviant among negative standards). In addition, reaction times to negative deviants should be slower than to positive deviants. If emotional changes have no impact on behavioral responses, then responses to the negative deviants should be inhibited regardless of the valence of the standards. Behavioral evidence, therefore, can provide a deeper understanding of the function of pre-attentive detection of emotional change in a temporal context.

The current study tested whether emotional changes in visual sequences resulted in vMMN and modulated behavioral responses using Japanese kanji with emotional connotations. These experiments can provide useful insight into how the automatic detection system for emotional changes functions at both the neural and behavioral levels.

## Experiment 1

We first tested whether emotional deviants within neutral standards elicited occipital negativity that was interpretable as vMMN and whether the amplitude of this negativity reflected the magnitude of the emotional changes in a sequence. One concern in regard to this paradigm was confounding results based on the physical differences between deviants and standards because vMMN reflects perceptual changes in visual features of the stimuli. To overcome this issue, all types of Japanese kanji consisting of standards and deviants were presented in the same frequency in a sequence. Therefore, vMMN for deviants could be derived from emotional changes rather than physical changes in a stimulus sequence because there was no infrequent stimulus in terms of physical features. We hypothesized that deviants with strong intensity would elicit larger negativities than would those with weak intensity when presented against neutral standards.

### Materials and Method

#### Participants

Twenty-seven adults participated in this study. They were recruited with advertisements placed by an intermediary company. Their occupational backgrounds varied widely, and all received compensation for participating. All participants had normal or corrected-to-normal vision and were native Japanese speakers. Data that contained excessive eye-blink artifacts were excluded from the analyses. In total, data from twenty-four participants were included in the final analysis (11 men and 13 women; aged 25.3±4.1 years).

#### Stimulus material

Stimuli consisted of individual Japanese kanji selected from a pool of 84 items. The Japanese kanji stimuli were rated in terms of valence on a nine-point scale (from extremely positive, 1, to extremely negative, 9) in a previous study [27]. The characters used in the experiment and their specifications are presented in Table 1. To verify differences in emotional intensity between emotionally strong, weak, and neutral stimuli, we tested the valence ratings of selected kanji. A one-way ANOVA revealed a main effect of stimulus type that was significant for the negative group (*F*(75, 150) = 107.0, *p*<.05) and the positive group (*F*(75, 150) = 97.2, *p*<.05). Multiple comparisons using the Tukey-HSD test showed that strongly and weakly negative deviants were significantly more unpleasant than neutral standards (*HSD* = 0.44, *α* = .05). The difference between strongly negative deviants and weakly negative deviants was marginally significant (*HSD* = 0.39, *α* = .10). For the positive group, strongly and weakly emotional deviants were rated as significantly more pleasant than neutral standards (*HSD* = 0.42, *α* = .05). There was a significant difference between strong and weak deviants of positive valence (*HSD* = 0.42, *α* = .05).

**Table 1:** Emotional word stimuli in Experiment 1.

					Valence	
Stimuli	Pronunciation	Meaning	Stroke Counts	Condition	*M*	*SD*
?	Bou	Hope	11	Strongly Positive Deviant	2.53	1.16
?	Ei	Glory	9	Weakly Positive Deviant	3.05	1.38
?	Satsu	Kill	10	Strongly Negative Deviant	7.54	1.70
?	Ki	Danger	6	Weakly Negative Deviant	7.13	1.53
?	Ka	Department	9	Neutral Standard	4.76	1.50
?	Jyo	Pact	7	Neutral Standard	4.82	0.99
?	Kaku	Stroke	8	Neutral Standard	4.88	1.03
?	Tei	Degree	12	Neutral Standard	4.97	1.02
?	Mai	Every	6	Neutral Standard	5.00	1.03
?	Zu	Figure	7	Neutral Standard	5.00	1.10
?	Han	Board	8	Neutral Standard	5.09	0.88
?	Kei	Plan	9	Neutral Standard	5.16	1.33

Note. Ratings were on a scale ranging from 1 (positive) to 9 (negative).

**Figure 1:**
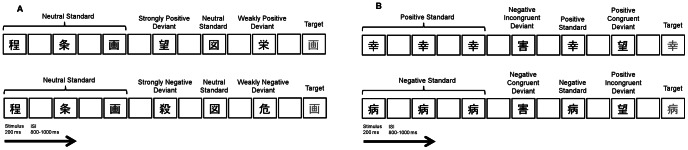
The sequence of stimuli for the two experimental paradigms. The duration of each stimulus was 200 ms. The inter-stimulus interval (ISI) was altered within 800–1000 ms. (A) The two sequences for Experiment 1. Strongly positive (negative) deviants and weakly positive (negative) deviants were infrequently presented in a sequence of neutral standards. All types of Japanese kanji consisting of standards and deviants were presented in the same frequency in the sequence. (B) The positive and negative sequences for Experiment 2. Positive deviants and negative deviants were infrequently presented in a sequence of standards. In the sequence of positive standards, positive deviants served as emotionally congruent deviants and negative deviants served as emotionally incongruent deviants. In the sequence of negative standards, negative deviants served as emotionally congruent deviants and positive deviants served as emotionally incongruent deviants.

We standardized the character configurations by using approximately the same number of strokes for each. All of the Japanese kanji were among the 1000 most-frequently used characters according to a survey on the use of Japanese kanji (http://sites.google.com/site/shibano/shin-jouyouJapanese kanji-hyou-no-tame-no-kanji-shutsugen-hindo-chousa). The experimental sequence is depicted in Figure 1A. To facilitate pre-attentive processing of emotional change, task-relevant stimuli (i.e., targets) were also presented. Whereas the standards and deviants were written in black ink, the targets, the same characters used for standards, were written in gray ink. The participants were asked to respond only to characters written in gray. The positive sequence consisted of neutral standards, two types of deviants (i.e., Bou (hope) for a strongly positive deviants and Ei (glory) for a weakly positive deviant) and targets. Negative sequences were arranged in the same manner (i.e., Satsu (kill) for a strongly negative deviant and Ki (danger) for a weakly negative deviant). All stimuli were displayed in a Gothic font on a white background.

#### Procedure

Experiments were conducted individually in an electronically shielded and sound-attenuated room. All participants provided their written informed consent to participate in the experiment. The Ethics Committee of RIKEN approved the informed consent procedure and the experimental protocol. After attaching all sensors, task instructions were provided.

The participants were seated in a comfortable chair and viewed the stimuli on a computer monitor. The viewing distance was 60 cm. Experimental events were controlled by a program written in Presentation version 14.1 (Neurobehavioral Systems, Inc.) and were run on a Dell Vostro 420 computer using the Microsoft Windows XP operating system. The visual stimuli were presented on a 17-inch monitor (PLE1702S, Iiyama; 1024 × 768 pixels, 32-bit color, 75-Hz refresh rate). The stimuli subtended a visual angle of approximately 6.8° × 7.2°.

The participants were asked to fixate on a character presented in the center of the screen and respond to a gray-colored target character as quickly and accurately as possible using a response key-pad with four buttons. They held the response key-pad with both hands and were allowed to use any button they chose. The response style was relatively unrestricted because we did not analyze reaction time in the ERP study. Although the response task forced the participants to pay attention to the stimuli sequence, responding to the gray-colored character took the participants' attention away from the emotional connotations of the stimuli. The positive sequence included positive deviants, and the negative sequence contained negative deviants. In each sequence, 160 neutral standards, 20 strongly emotional deviants, 20 weakly emotional deviants, and 16 targets were presented in random order. Neutral standards consisted of eight different types of characters. Therefore, all characters belonging to standards or deviants were presented the same number of time (i.e., twenty times), and each target corresponding to each standard was displayed twice. Deviants were never presented consecutively. All stimuli were presented for 200 ms and the inter-stimulus interval was randomly varied from 800 ms to 1000 ms. Each block, which included four successive positive or negative sequences, was administered once. The order of the positive and negative blocks was counterbalanced among the participants, and there were short breaks between the sequences and blocks.

#### Data recording and analysis

Electroencephalograms (EEGs) were continuously recorded with Ag-AgCl electrodes located at FZ, F3, F4, F7, F8, FCz, FC3, FC4, FT7, FT8, CZ, C3, C4, T7, T8, CPz, CP3, CP4, TP7, TP8, Pz, P3, P4, P7, P8, Oz, O1, and O2 (10–20 system), online band-pass filtered from 0.15 to 30 Hz, and sampled at 500 Hz using a Scan 4.2 acquisition system (SynAmps; Neuro-Scan). All electrodes on the scalp were referenced to an electrode on the tip of the nose. Electrode impedance was maintained at or below 10 kΩ. Horizontal and vertical electrooculograms (EOGs) were recorded simultaneously with bipolar derivation to eliminate time periods contaminated by eye-movement artifacts.

EEG data were analyzed using the EDIT module of Scan 4.2. The continuous recording data were cut into 700-ms time periods (100 ms pre-stimulus to 600 ms post-stimulus) with the 100-ms pre-stimulus set as the baseline. EEG data for targets was not included in the analysis because the targets were prepared to draw attention away from emotional change in the visual sequence. First, data containing amplitude changes of over 100 µV for EEG channels and time periods were automatically rejected. Next, an experimenter scanned the data to detect any additional disturbances. The numbers of rejected trials for the final participants did not differ between the standards (*t*(23) = 0.25, *n.s.*) or among the deviants (*F*(1,23) = 0.20, *n.s.*). EEG waves were averaged for each participant within each condition. To ensure there were corresponding numbers of standards and deviants, only the standards presented just prior to the deviants were used in the analysis. The grand average across all participants under each condition was also computed.

**Figure 2:**
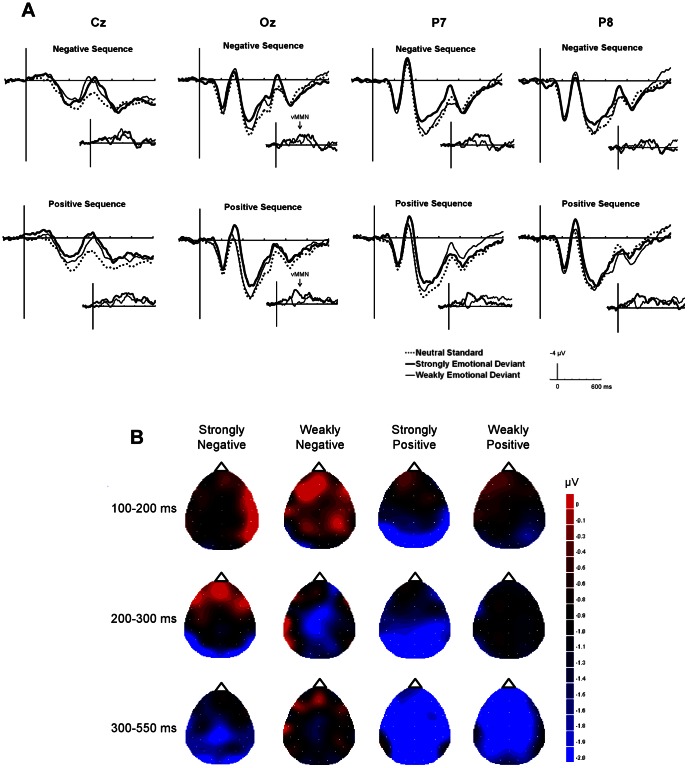
ERP results in Experiment 1. (A) The grand-average ERPs at Cz, Oz, P7, and P8 elicited by standards (dashed line), strongly emotional deviants (bold line), and weakly emotional deviants (thin line) in negative and positive sequences. The inset shows vMMN, grand average deviant-minus-standard, for strongly emotional deviants (bold line) and weakly emotional deviants (thin line). (B) The topographies of the voltage difference between the standards and the deviants at the peak latencies for each time period. The images have been flattened into a two-dimensional space using the global interpolation method, which uses all electrodes to calculate values at any given point, viewed from the top of the head.

The measurement windows for the ERP amplitudes were determined based on prior literature and visual inspection of the grand-averaged wave forms. This visual inspection revealed that negative shifts for deviants relative to standards occurred at 100–400 ms in the negative sequence and at 100–550 ms in the positive sequence (see Figure 2A). For the amplitude analysis, ERPs at 100–550 ms were divided into three time periods. First, a time window of 100–200 ms was extracted as an initial time period. Next, EPN, which is thought to reflect the emotional significance of stimuli, can be observed at 150-ms post-stimulus and prominently at a latency of 200–300 ms [7], [32]. Therefore, the range between 200–300 ms was extracted as a middle time period to examine the influence of negativity elicited by emotional significance of deviants. Lastly, the range between 300–550 ms was extracted as a late time period. In the late time period, it is possible to observe P3, which has been elicited in an oddball task. For all time periods, mean voltages were computed across three occipital electrodes (O1, O2, and Oz) where vMMN should appear pronounced [11], [18], [33]. For the initial and middle time periods, as EPN is usually observed in posterior regions, two posterior electrodes over the right hemisphere (TP8 and P8) and two homologous electrodes over the left hemisphere (TP7 and P7) were averaged for each. Mean amplitude in the late time period was measured across three centro-parietal electrodes (FCz, Cz, and CPz) where P3 should be pronounced. The mean amplitude of the ERPs was analyzed using a three-way repeated-measures ANOVA with variables for sequence (positive, negative), stimulus type (standards, strongly emotional deviants, and weakly emotional deviants), and electrode site (occipital, right, and left for the latencies of 100–200 ms and 200–300 ms; occipital and centro-parietal for the latency of 300–550 ms) as factors. Because a main goal of this study is to examine whether there is significant ERP difference between the responses to the standards and the deviants (i.e., the emergence of vMMN), only the interactions and the main effects concerning stimulus type will be further analyzed. The Greenhouse-Geisser adjustment was applied, and corrected *p* values are reported along with uncorrected degrees of freedom.

### Results

Figure 2A shows the grand-averaged ERPs elicited at Oz (occipital area), P8 (right posterior), P7 (left posterior), and Cz (centro-parietal) electrodes by neutral standards, strongly emotional deviants, and weakly emotional deviants in the positive and negative sequences and the difference in ERP waves between the deviants and the standards at each site. ERP waves at Oz were greater for strongly emotional deviants than for weakly emotional deviants, especially during the 200- to 400-ms period following a negative deviant and the 150- to 320-ms period following a positive deviant.

The topographical distributions of the differential amplitudes at the peak latency are shown in Figure 2B. Negativities were observed at the occipital and posterior scalp locations for all stimuli at the latency of 100–200 ms and for negatively strongly emotional deviants at the latency of 200–300 ms. Negativities were distributed across all scalp locations for weakly negative and strongly positive deviants at the latency of 200–300 ms and for strongly negative and both strongly and weakly positive deviants at the latency of 300–550 ms.

**Figure 3:**
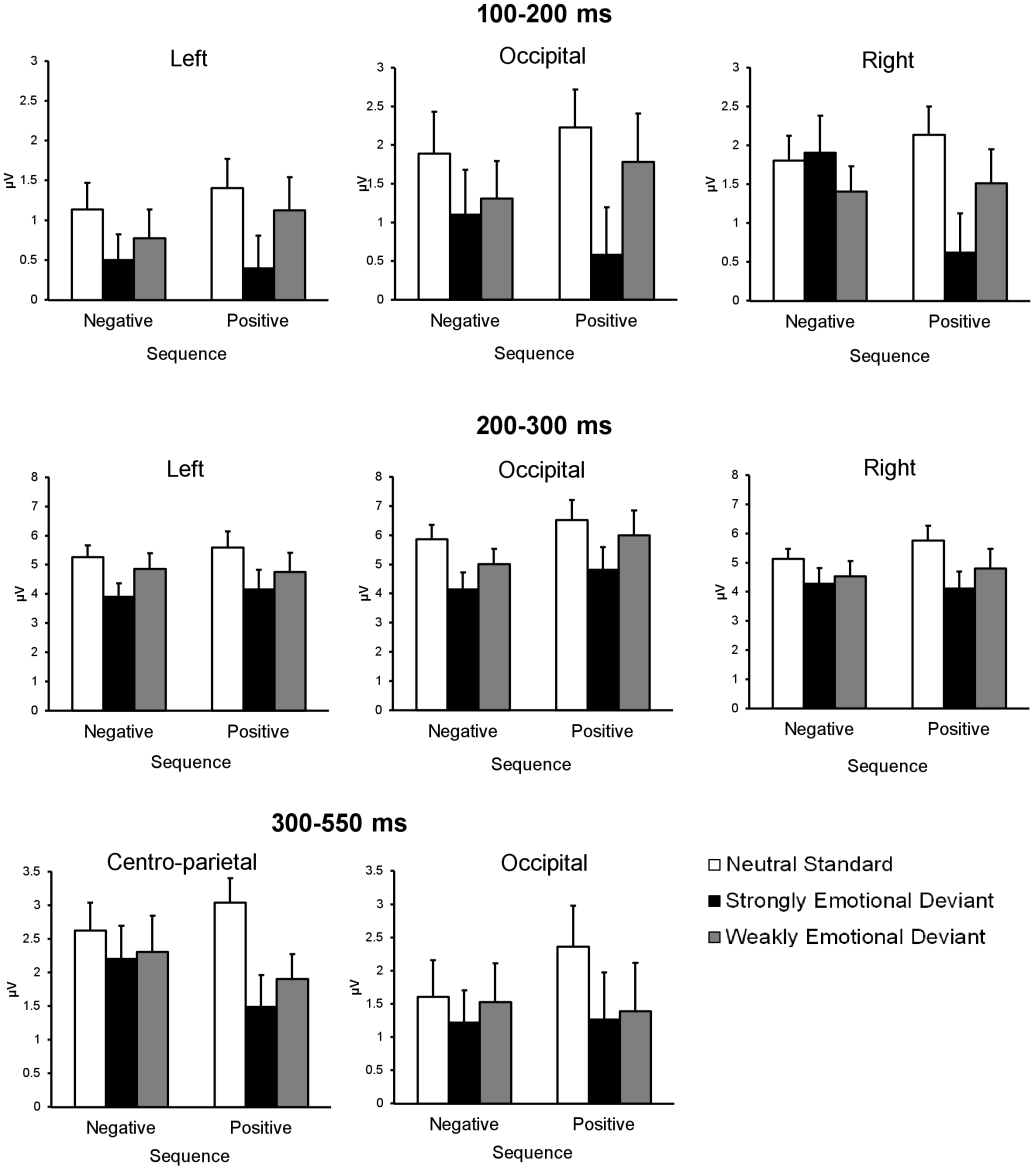
Mean amplitudes and SEMs of ERPs for each condition. ERP amplitudes for neutral standards, strongly emotional deviants, and weakly emotional deviants in negative and positive sequences. For all of the time windows, ERP amplitudes were averaged across three occipital electrodes (O1, O2, and Oz). In addition, for 100–200 ms and 200–300 ms, ERP amplitudes were averaged across two posterior electrodes over the right hemisphere (TP8 and P8), and two homologous electrodes over the left hemisphere (TP7 and P7). For 300–550 ms, ERP amplitudes were averaged across three centro-parietal electrodes (FCz, Cz, and CPz).

Figure 3 shows the mean amplitude of the grouped electrode sites for the 100–200 ms, 200–300 ms, and 300–550 ms intervals used for the ANOVAs. A three-way ANOVA for the ERP amplitudes at 100–200 ms revealed a significant main effect of stimulus type (*F*(2,46) = 7.54, *p*<.01). Multiple comparisons using the Tukey-HSD test showed that the amplitude for strongly emotional deviants shifted more negatively than that for standards (*HSD* = 0.66, *α* = .05). A significant three-way interaction was also found (*F*(4,92) = 3.14, *p*<.05), indicating three significant simple interactions (sequence × stimulus type site for the right hemisphere: *F*(2,138) = 5.63, *p*<.01; sequence × electrode site for the strong deviants: *F*(2,138) = 10.04, *p*<.01; stimulus type × electrode site for the negative sequence: *F*(4,184) = 3.77, *p*<.01). The significant simple-simple main effects yielded by these interactions revealed three main findings. First, the strongly positive deviants elicited more negative-going ERP waves than the standards or the weakly positive deviants in the right hemisphere (*F*(2,276) = 13.88, *p*<.01) (*HSD = *0.72, *α = *.05). Second, only in the strong condition, the positive deviants evoked more negativity than the negative deviants in the right hemisphere (*F*(1,207) = 12.23, *p*<.01). Lastly, strongly negative deviants elicited more negativity at the left hemisphere than at the right hemisphere (*F*(2,276) = 8.32, *p*<.01) (*HSD = *0.68, *α = *.05).

At a latency of 200–300 ms, the main effect of stimulus type reached significance (*F*(2,46) = 12.65, *p*<.01), indicating that strongly emotional deviants elicited more negative-going waveforms than the standards did at all the electrode sites (*HSD = *0.84, α = .05).

At a latency of 300–550 ms, only a main effect of stimulus type was significant (*F*(2,46) = 3.33, *p*<.05), indicating that the ERP waveform was significantly more negative-going for strongly emotional deviants than for standards at all electrode sites (*HSD = *0.68, *α = *.05).

### Discussion

Deviant stimuli of strong and weak emotional intensity were presented infrequently among neutral standards. At the latencies of 200–300 ms and 300–550 ms, a greater negative shift was observed in the occipital regions for strongly emotional deviants than for neutral standards. This result is consistent with previous studies that used emotional facial expressions presented as deviants within a series of neutral faces [19], [20]. In the current study, the observed occipital negativity could be regarded as a possible vMMN for emotional change in a sequence.

Weakly emotional deviants elicited equivalent ERP amplitudes compared to standards, although these weak deviants were still reported by psychological ratings as more pleasant or unpleasant than neutral standards. Weakly emotional deviants might be insufficient to evoke vMMNs for emotional changes because of their lower emotional intensity. It is possible that vMMNs for emotional changes are not evoked until the magnitude of emotional change in a visual sequence reaches a threshold for detection, instead of reflecting the magnitude of emotional change.

At the latency of 100–200 ms, the negative shift elicited by the strongly positive deviants was prominent in the right posterior region, a potential generation source of EPN [7], [8]. This result suggests that the standard-deviants difference at the initial latency was derived from the emotional significance of deviants, not emotional change itself. This is inconsistent with the results of previous studies demonstrating that vMMN for facial expressions emerged from the latencies of 110 ms [20] and 150 ms [19]. It is possible that the initial part of vMMN in response to emotional change might be specific for facial expressions.

Furthermore, at the latency of 100–200 ms, the right hemisphere was sensitive to the emotional intensity of positive stimuli and to the valence category. Given that the current task assessed pre-attentive emotional processing, the results are in line with the fact that the right hemisphere is characterized by unconscious processing of emotion, unawareness and automaticity [34]. However, the finding that negativity elicited by strongly negative deviants was greater in the left hemisphere than in the right hemisphere is inconsistent with the facts mentioned above. Further research is needed to determine whether hemispheric differences emerge in the emotional processing of a visual sequence.

In summary, the greater occipital negativity for strongly emotional deviants than for neutral standards was identified as a vMMN for emotional change. However, enhanced negativity by strongly emotional deviants was also observed in the right and left posterior and centro-parietal areas in the latencies of 200–300 ms and 300–550 ms. Hence, it is possible that the negativity for strongly emotional deviants was contaminated by EPN. To clarify this issue, Experiment 2 confirms whether the negativity derived from emotional deviants 200 ms after stimulus presentation can be regarded as vMMN by using an elaborated experimental paradigm.

## Experiment 2

To differentiate the negative shift elicited by emotional change from that elicited by the emotional significance of deviants, a single character was presented as two types of deviants depending on the valence of standards. One was emotionally incongruent with the standard (i.e., positive deviants against negative standards), and the other was emotionally congruent with the standard (i.e., positive deviants against positive standards). If the negative shift in ERP waves for emotionally incongruent deviants was greater than that for emotionally congruent deviants, then this difference would be derived from the emotional change in the visual sequence rather than from the emotional significance of the deviants because the two deviants were the same character. Furthermore, the sequential regularities were established by not only the emotional aspect but also the perceptual aspect of the standards because only one character served as the standard in a sequence. Hence, the character configurations of both types of deviants perceptually violated the representation of the sequential regularities acquired by the standards. So if the emotionally incongruent deviants and the emotionally congruent deviants equally increased occipital negativity compared to the standards, the negativity could be interpreted as vMMN in response to the differences in visual features between the deviants and the standards.

### Materials and Method

#### Participants

Twenty-five adults participated in the study. They were recruited with advertisements placed by an intermediary company, and their occupational backgrounds varied widely. All received compensation for participating in the experiment. All participants were native Japanese speakers and had normal or corrected-to-normal vision. Data containing excessive eye-blink artifacts were excluded from the analyses. Data from eighteen participants were used in the final analysis (nine men and nine women; aged 28.5 ± 6.1 years).

#### Stimulus material

Stimuli were selected from the pool of Japanese kanji used in Experiment 1. The Japanese kanji used in Experiment 2 and their specifications are presented in Table 2. To confirm whether the valence ratings for standards and deviants were equivalent, two-tailed *t*-tests were conducted for both positive and negative stimuli. For both positive and negative stimuli, there were no significant differences of valence ratings between standards and deviants (positive: *t*(75) = 0.33, *n.s.*; negative: *t*(75) = 0.18, *n.s.*). The experimental sequence is depicted in Figure 1B. There were two types of visual sequences; a positive sequence consisted of positive standards and a negative sequence consisted of negative standards. An identical Japanese kanji served as an emotionally congruent or incongruent deviant depending on the valence of the standards. For example, the positive deviant (Bou: hope) was emotionally congruent with the standard in the positive sequence (Kou: happiness) but incongruent with the standard in the negative sequence (Byo: sickness). Conversely, the negative deviant (Gai: harm) was emotionally congruent with the standard in the negative sequence (Byo: sickness) but not in the positive sequence (Kou: happiness). Both sequences included task-relevant stimuli (i.e., targets). Whereas standards and deviants were presented in black ink, targets were the same characters as used for standards but written in gray ink. All stimuli were displayed in a Gothic font on a white background.

**Table 2:** Emotional word stimuli in Experiment 2.

					Valence	
Stimuli	Pronunciation	Meaning	Stroke Counts	Condition	*M*	*SD*
?	Kou	Happiness	8	Positive Standard	2.58	1.63
?	Bou	Hope	11	Positive Deviant	2.52	1.16
?	Byo	Sickness	10	Negative Standard	7.40	1.58
?	Gai	Harm	10	Negative Deviant	7.43	1.53

Note. Ratings were on a scale ranging from 1 (positive) to 9 (negative).

#### Procedure

All participants provided their written informed consent to participate in the experiment. The Ethics Committee of RIKEN approved the informed consent procedure and the experimental protocol. The presentation setting for the task was identical to that used in Experiment 1. Each sequence was composed of 200 standards, 20 emotionally congruent deviants, 20 emotionally incongruent deviants, and 20 targets. The participants were asked to respond to a gray-colored target character as quickly and accurately as possible. No deviants were presented consecutively, and deviants were always preceded by standards. The time windows for stimulus presentation and implementation of sequences and blocks were identical to those in Experiment 1.

#### Data recording and analysis

Data recording and analysis were conducted identically to the procedures followed in Experiment 1. The numbers of rejected trials for the remaining participants did not differ between the standards (*t*(17) = 0.37, *n.s.*) or among the deviants (*F*(1,17) = 3.15, *n.s.*).

**Figure 4:**
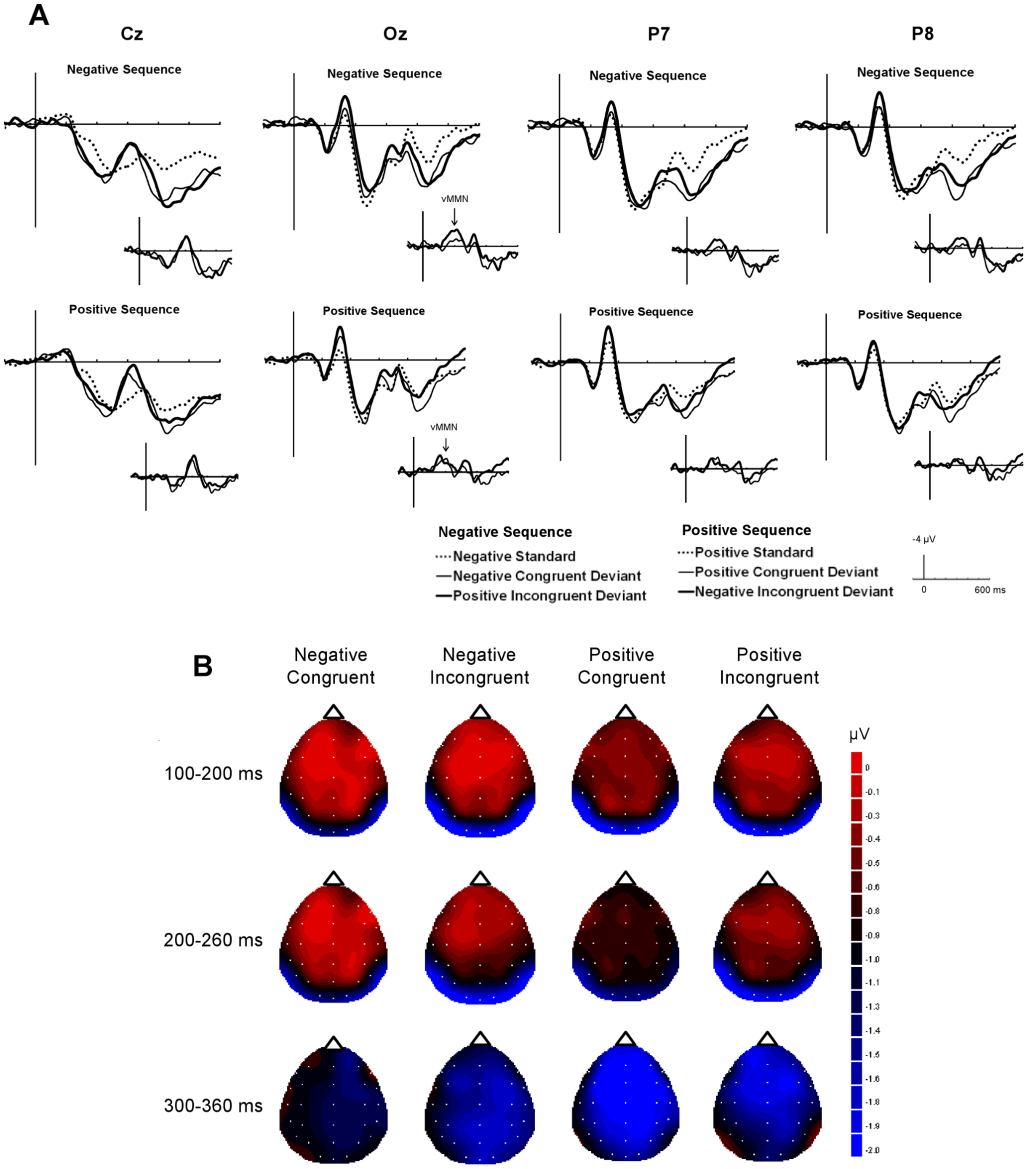
ERP results in Experiment 2. (A) The grand-average ERPs at Cz, Oz, P7, and P8 elicited by standards (dashed line), emotionally incongruent deviants (bold line), and emotionally congruent deviants (thin line) in negative and negative sequences. The inset shows vMMN, grand average deviant-minus-standard, for emotionally incongruent deviants (bold line) and emotionally congruent deviants (thin line). (B) The topographies of the voltage difference between the standards and the deviants at the peak latencies for each time period. The calculations for the differential ERP waves between standards and deviants were performed within each sequence (i.e., the ERP amplitude for positive standards was subtracted from that for incongruent negative deviants and congruent positive deviants). The images have been flattened into a two-dimensional space using the global interpolation method, which uses all electrodes to calculate values at any given point, viewed from the top of the head.

The time window for statistical analysis was determined in the same manner as Experiment 1. Visual inspections of the grand-averaged waves at Oz revealed that negative shifts for deviants were observed from 100 to 260 ms and from 300 to 360 ms (see Figure 4A). These time windows were divided into three time periods: 100–200 ms, 200–260 ms, and 300–360 ms. For all time periods, mean voltages were computed across three occipital electrodes (O1, O2, and Oz). For the latencies of 100–200 ms and 200–260 ms where the EPN is prominent, amplitudes were averaged from two posterior electrodes over the right hemisphere (TP8 and P8) and two homologous electrodes over the left hemisphere (TP7 and P7). Mean amplitude at the latency of 300–360 ms, where P3 might emerge, was measured across three centro-parietal electrodes (FCz, Cz, and CPz). The mean amplitude of the ERPs was analyzed by a three-way repeated-measures ANOVA using the following factors: stimulus emotion (positive, negative), stimulus type (standards, emotionally congruent deviants, and emotionally incongruent deviants), and electrode site (occipital, right, and left for the latencies of 100–200 ms and 200–260 ms; occipital and centro-parietal for the latency of 300–360 ms). Only interactions and main effects including the factor of stimulus type will be further analyzed as well as in Experiment 1. The Greenhouse-Geisser adjustment was applied.

### Results

Figure 4A shows the grand-averaged ERPs elicited at Oz (occipital area), P8 (right posterior), P7 (left posterior), and Cz (centro-parietal) electrodes by standards, emotionally congruent deviants, and emotionally incongruent deviants in the positive and negative sequences and difference in ERP waves between the deviants and the standards at each site. The ERP waves at Oz for the two types of deviants shifted in the negative direction compared with those for the standards in both the positive and negative sequences.

The topographical distributions of the differential amplitudes at the peak latency between standards and deviants are shown in Figure 4B. Occipital and posterior negativities were observed at the peak latencies of 100–200 ms and 200–260 ms. For the peak latency of 300–360 ms, negativity emerged from the frontal to central region for all stimuli.

**Figure 5:**
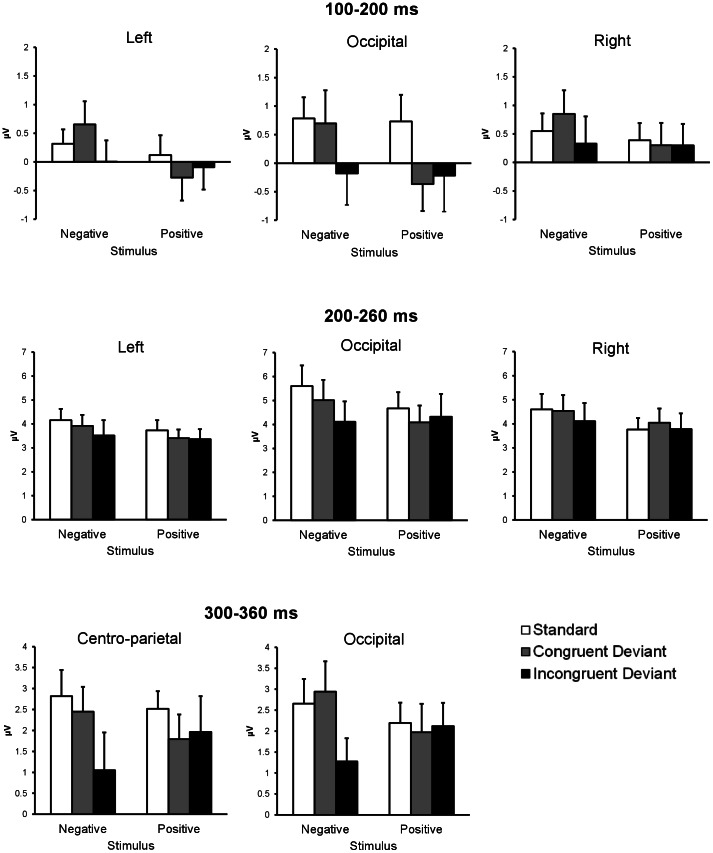
Mean amplitudes and SEMs of ERPs for each condition. ERP amplitudes for standards, emotionally congruent deviants, and emotionally incongruent deviants in negative and positive sequences. For all of the time windows, ERP amplitudes were averaged across three occipital electrodes (O1, O2, and Oz). In addition, for 100–200 ms and 200–260 ms, ERP amplitudes were averaged across two posterior electrodes over the right hemisphere (TP8 and P8), and two homologous electrodes over the left hemisphere (TP7 and P7). For 300–360 ms, ERP amplitudes were averaged across three centro-parietal electrodes (FCz, Cz, and CPz).

Figure 5 shows the mean amplitudes of the grouped electrode sites for 100–200 ms, 200–260 ms, and 300–360 ms intervals used for the ANOVAs. At a latency of 100–200 ms, there was a significant stimulus type × electrode site interaction (*F*(4, 68) = 9.57, *p*<.01), indicating that emotionally incongruent and congruent deviants elicited significantly greater negativity than the standards did in the occipital area only (*F*(2, 102) = 6.32, *p*<.01) (*HSD* = 0.65, *α* = .05). No other significant main effects or interactions were found.

At a latency of 200–260 ms, only the stimulus type × electrodes site interaction was significant (*F*(4, 68) = 4.81, *p*<.01), showing that the negativity was significantly greater for the emotionally incongruent deviants than for the standards in the occipital area only (*F*(2, 102) = 3.80, *p*<.05) (*HSD = *0.80, *α = *.05). No other main effects or interactions were significant.

At a latency of 300–360 ms, a main effect of stimulus type was significant (*F*(2, 34) = 3.58, *p*<.05), indicating that the emotionally incongruent deviants elicited significantly greater negativity than the standards did at all the electrode sites (*HSD = *0.72, *α = *.05). No other main effects or interactions were significant.

### Discussion

At the latency of 200–260 ms, emotionally incongruent deviants, but not congruent deviants, elicited more occipital negativity than standards. The results indicate that the difference in ERP amplitudes between emotionally incongruent deviants and standards was derived from emotional change. Although emotionally incongruent and congruent deviants had identical emotional connotations, emotionally congruent deviants elicited no occipital negativity compared to standards. The results suggest that occipital negativity elicited by emotionally incongruent deviants was not derived from the emotional significance of the deviants. This is evidence, therefore, that ERP amplitudes can reflect emotional change in a visual sequence at the latency of 200–260 ms. In addition, significant differences in mean amplitude between emotionally incongruent deviants and standards were observed only in the occipital area. Considering that the vMMN is predominantly observed in the occipital area [33], the negativity elicited by emotionally incongruent deviants is a vMMN representing emotional change. Although the EPN response may have contaminated the negativity, the EPN is more pronounced at the posterior area than the occipital area [7], [8]. Hence, these negativities can most likely be attributed to vMMN for emotional change in a visual sequence.

At the latency of 100–200 ms, emotionally congruent and incongruent deviants elicited equivalent occipital negativity that was greater than the negativity elicited by the standards. Given that both types of deviants showed physically different configurations compared to the standards, the visual features of the deviant stimuli perceptually violated the sequential regularities established by the repetition of the same characters (i.e., the standard stimuli). Thus, the observed occipital negativities at the latency of 100–200 ms might reflect the changes in the visual features of the stimuli, rather than the emotional connotations.

At the latency of 300–360 ms, emotionally incongruent deviants elicited greater negativity than standards at the occipital posterior and centro-parietal areas. This finding is inconsistent with the fact that the positive component P3 can be elicited by infrequent emotional stimuli in an oddball task [7]. This discrepancy could be due to task demands. Although the P3 response can be enhanced when infrequent stimuli are task-relevant and focused upon [35], the emotional deviants in this experiment were pre-attentively processed. Hence, emotionally incongruent deviants elicited negativity rather than positivity even at 300 ms post-stimulus. It is still unclear whether this negativity can be attributed to a vMMN because the negativities were observed not only in occipital but also in centro-parietal areas. Further research is needed to investigate whether the late negativity elicited by emotional deviants relates to the vMMN.

We found that brain signals (i.e., vMMN) can reflect the processing of an emotional change within a visual sequence. However, if emotional changes occur in a temporal context, organisms take action in response to incoming novel stimuli. In Experiment 3, we examined whether emotional changes would modulate behavioral responses using vMMN methodology identical to that used in Experiment 2.

## Experiment 3

Experiment 3 investigated whether the pre-attentive detection of emotional change would be reflected in behavioral data measured in terms of reaction time. In Experiment 3, the emotional changes in the sequence were task-irrelevant as well as in the ERP studies. We posed two hypotheses. First, if emotional change modulated behavioral responses, the reaction time to emotionally incongruent deviants would be faster than that to emotionally congruent deviants. Second, as for a valence effect of emotional deviants, the reaction time to negative deviants would be slower than that to positive deviants.

### Materials and Method

#### Participants

Twenty-eight adults participated in the study (eight men and 20 women; aged 31.4 ± 7.9 years). The participants were staff members at RIKEN and had not participated in Experiment 1 or 2. All had normal or corrected-to-normal vision and were native Japanese speakers. All participants provided their written informed consent to participate in the experiment. The Ethics Committee of RIKEN approved the informed consent procedure and the experimental protocol.

#### Stimulus materials

The set of stimuli was identical to that used in Experiment 2 with minor changes made to the stimulus colors. Both the deviants and the standard stimuli that acted as targets for participant responses were presented in gray.

#### Procedure and data analysis

The experiment was conducted by a program written in Inquisit 3.0 (Millisecond) and was implemented on a Dell Vostro 420 computer using the Microsoft Windows XP operating system. The presentation setting, the time windows for stimulus presentation, and implementation of sequences were identical to those in Experiment 2. The stimuli were presented in a positive sequence that included positive standards and a negative sequence that included negative standards. In each sequence, 100 standards, 10 emotionally congruent deviants, and 10 emotionally incongruent deviants were randomly presented in black ink. Ten standards, 10 emotionally congruent deviants, and 10 emotionally incongruent deviants were randomly presented as targets in gray ink. Each sequence was administered once. The participants were asked to detect a gray target by pressing the “5” key as quickly and accurately as possible on a numeric keypad. The keypad was placed on the desk, and the participants responded using the first finger of their dominant hand. Reaction time was defined as the time from stimulus onset to participant response. We analyzed reaction time data for target stimuli consisting of standards, emotionally incongruent deviants, and emotionally congruent deviants.

### Results

**Figure 6:**
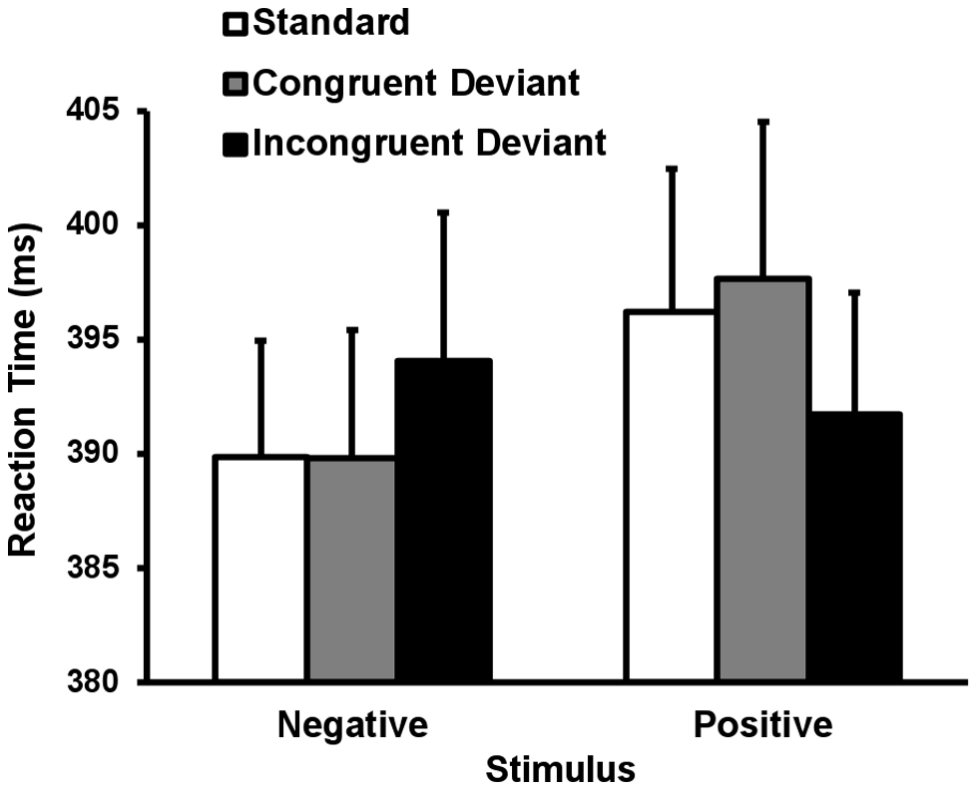
Mean reaction times and SEMs for each condition.

Accuracy rates for target detection were fairly high (*M = *99.84%, *SD = *0.40). Figure 6 shows the mean reaction times across conditions. A two-way repeated-measures ANOVA on reaction times was conducted with target emotion (positive, negative) and stimulus type (standard, emotionally congruent deviants, and emotionally incongruent deviants) as factors. Greenhouse–Geisser corrections were not needed. The main effect of target emotion was significant, indicating that the reaction times for negative targets were faster than those for positive targets regardless of stimulus type (*F*(1, 27) = 6.09, *p*<.01). There was no significant main effect of stimulus type or interaction.

### Discussion

The results revealed no significant differences in reaction time for the standards, emotionally incongruent deviants, and emotionally congruent deviants. This suggests that, contrary to the ERP results in Experiment 2, emotional changes in the visual sequence did not modulate behavioral responses. Importantly, negative stimuli facilitated behavioral responses regardless of the valence of standards. One possible explanation is that the detection of threat stimuli without regard to context is crucial to taking action against them, i.e., fight or flight. Hence, detection of the emotional significance of negative stimuli is more important than detection of emotional change in the environment at the behavioral level.

However, our results are inconsistent with the fact that behavioral responses to negative stimuli are inhibited compared to the responses to neutral and positive stimuli [30], [31]. This inconsistency might be due to the difference in the overt task between the current study and the previous studies. The previous studies used the cognitive task in which participants were required to scan stimuli, per se, to evaluate emotion-irrelevant aspects of the stimuli (i.e., the length of the nose in emotional facial expressions). Therefore, negative contents of the stimuli capture their attention, resulting in increased reaction time to the stimuli. On the other hand, the current task required the participants to respond just to color changes within a visual sequence. The color-change detection task is less complex and requires less attention to the relevant stimuli than the cognitive task does. It is possible that subsequent behavioral responses to negative stimuli are facilitated when the over task requires less cognitive processing. Future research is needed to investigate behavioral responses to emotional stimuli within the temporal visual sequence.

## General Discussion

Across two ERP experiments, the present study investigated whether the vMMN reflects emotional changes in a sequence using Japanese kanji. In Experiment 1, we found a greater occipital negativity in response to strongly emotional deviants compared to that for neutral standards at latencies of 200–300 ms and 300–550 ms. This finding is consistent with previous studies using emotional facial expressions [19], [20]. However, it is possible that the negative shifts for strongly emotional deviants were driven by the emotional significance of the stimuli themselves. To separate EPN for the emotional significance of deviants from vMMN for emotional change, a single character was presented as two types of deviants; emotionally incongruent deviants and emotionally congruent deviatns. Those results showed that emotionally incongruent deviants, and not emotionally congruent deviants, elicited greater occipital negativities than standards at latencies of 200–260 ms and 300–360 ms. These results provide evidence that the vMMN is elicited by emotional change within a visual sequence. Furthermore, the similarity between the topographies in Experiment 1 and Experiment 2 suggests that the occipital negativities observed in the two experiments generally reflected an automatic evaluation system that detects emotional deviancy within a sequence.

The vMMN for emotional change was observed in the occipital area. Given that the neural generation of vMMN occurs in occipital visual extrastriate areas [36], [37], it is possible that the processing of emotional change can engage the visual extrastriate areas. This is supported by the presence of a vast amount of neural connections between the visual extrastriate areas and the amygdala, which is involved in the rapid processing of emotional stimuli [38]. Hence, it is possible that the activation of the amygdala in response to emotional change stimulates the visual extrastriate areas, thereby producing vMMN.

We found no differences in relation to valence between ERP amplitudes or latencies of vMMN for emotional change. The neural processing of emotional change does not appear to be modulated by directional valence shifts (i.e., from positive to negative or from negative to positive). A recent study of autonomic responses to emotional stimuli found that skin conductance responses (SCR) to negative pictures preceded by positive pictures were equivalent with those to positive pictures preceded by negative pictures [39]. Although SCR reflects sympathetic arousal level, the finding that the direction of valence change had no influence on physiological activity is consistent with our results. Taken together, physiological responses to emotional changes within a sequence might be insensitive to the valence of stimuli. Responses to emotional changes should be further tested using additional physiological measurement indices in future research.

Contrary to the findings of the ERP experiment, behavioral responses were not modulated by emotional change. The result indicated that reaction times for negative targets were facilitated compared to those for positive targets, regardless of emotional changes. In the behavioral experiment, participants were required to “respond,” per se, to the targets embedded in the emotional context. Hence, the negative content of the targets facilitates behavioral responses to address threatening situations immediately. On the other hand, emotional change is predominantly processed in the brain to detect the incoming novel stimuli in the environment.

Although this study expands the existing literature on the vMMN for emotional changes in a sequence, some limitations must be considered. First, this study used a small set of Japanese kanji stimuli. One concern is that the Japanese kanji used in the current study had emotional connotations with biological significance (i.e., harm and sickness). Given that the emotional processing of biologically emotional stimuli is physiologically different from that of socially emotional stimuli [23], [24], a set of Japanese kanji with a variety of emotional connotations should be used to investigate vMMN for emotional changes. In addition, another stimulus category (e.g., emotional pictures) needs to be used to generalize vMMN to emotional changes. Second, it remains unclear whether vMMN for emotional change occurs regardless of directional change in a sequence. In this study, the emotional deviants embedded within neutral standards (Experiment 1), positive deviants embedded within negative standards, and negative deviants embedded within positive standards (Experiment 2) were tested. Automatic detection of these deviants in the environment is important for organisms' survival. If vMMN facilitates this adaptive behavior, neutral deviants embedded within emotional standards could elicit little vMMN.

In summary, the present study yielded two major findings. First, we found that the vMMN is sensitive to emotional changes in a visual sequence consisting of Japanese kanji with emotional connotations. An elaborated experimental paradigm in Experiment 2 revealed that the vMMN for an emotional change occurred at the latencies of 200–260 ms and that the vMMN for a visual perceptual change emerged at the latency of 100–200 ms. Second, contrary to the results of the ERP experiments, emotional change had no influence on reaction time in an identical task to that used in the ERP experiment. Negative stimuli are dominantly detected regardless of context on a behavioral level. Future research is needed to investigate how the neural activities in response to emotional change modulate behavior in a temporal context.
