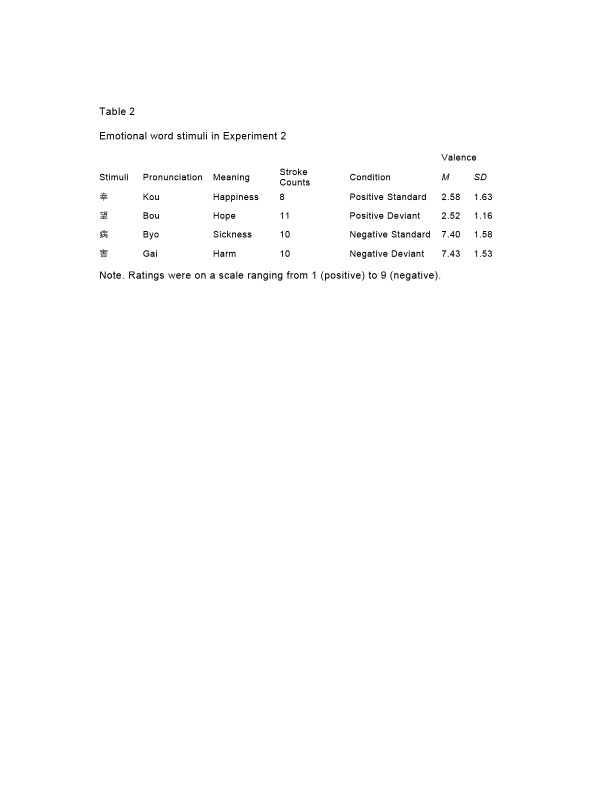# Correction: Event-Related Potentials Elicited by Pre-Attentive Emotional Changes in Temporal Context

**DOI:** 10.1371/annotation/d92eb257-5d10-4e61-8210-44c5cdf5d896

**Published:** 2013-09-25

**Authors:** Tomomi Fujimura, Kazuo Okanoya

There was an error in Table 1 and Table 2 Some characters erroneously appear as question marks. Please see the corrected Tables below.

Table 1: 

**Figure:**
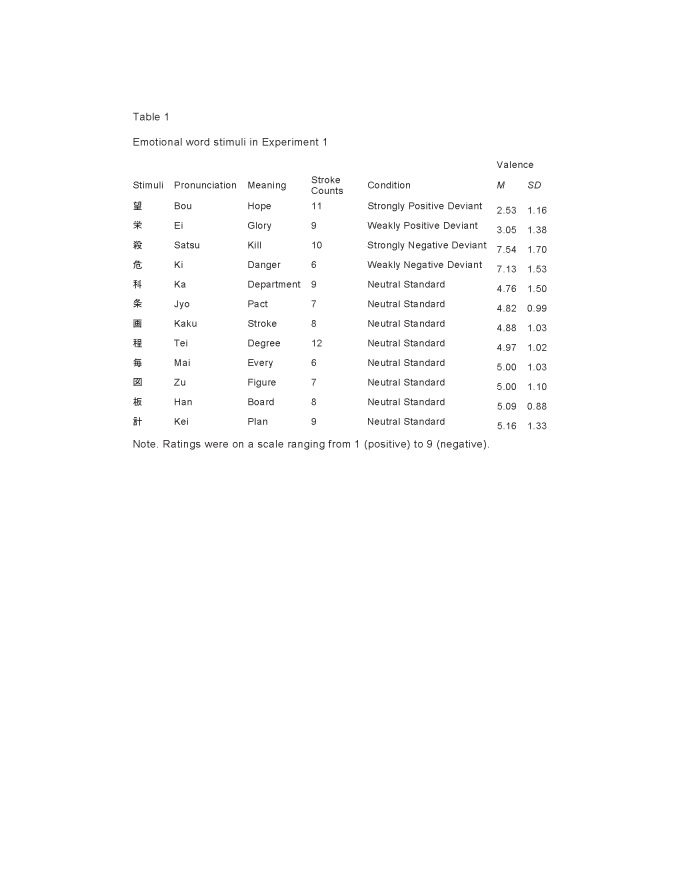


Table 2: 

**Figure:**